# Cerebrospinal fluid cell-free mitochondrial DNA is associated with HIV replication, iron transport, and mild HIV-associated neurocognitive impairment

**DOI:** 10.1186/s12974-017-0848-z

**Published:** 2017-03-31

**Authors:** Sanjay R. Mehta, Josué Pérez-Santiago, Todd Hulgan, Tyler R. C. Day, Jill Barnholtz-Sloan, Haley Gittleman, Scott Letendre, Ronald Ellis, Robert Heaton, Stephanie Patton, Jesse D. Suben, Donald Franklin, Debralee Rosario, David B. Clifford, Ann C. Collier, Christina M. Marra, Benjamin B. Gelman, Justin McArthur, Allen McCutchan, Susan Morgello, David Simpson, James Connor, Igor Grant, Asha Kallianpur

**Affiliations:** 1 Department of Medicine, University of California-San Diego, San Diego, CA USA; 2 Department of Medicine, San Diego Veterans Affairs Medical Center, San Diego, CA USA; 3 Department of Psychiatry, University of California, San Diego, CA USA; 4 Department of Medicine, Division of Infectious Diseases, Vanderbilt University, Nashville, TN USA; 5 Division of Biostatistics, Washington University, St. Louis, MO USA; 6 Case Comprehensive Cancer Center, Case Western Reserve University School of Medicine, Cleveland, OH USA; 7 Department of Neurology, University of California-San Diego, San Diego, CA USA; 8 Department of Neurosurgery, Pennsylvania State/Hershey College of Medicine, Hershey, PA USA; 9 Department of Neurology, Washington University, St. Louis, MO USA; 10 Department of Medicine, University of Washington, Seattle, WA USA; 11 Department of Neurology, University of Washington, Seattle, WA USA; 12 Department of Pathology, University of Texas Medical Branch, Galveston, TX USA; 13 Department of Neurology, Johns Hopkins University, Baltimore, MD USA; 14 Department of Neurology, Icahn School of Medicine at Mount Sinai, New York, NY USA; 15 Genomic Medicine Institute/Lerner Research Institute Cleveland Clinic, Cleveland, OH USA; 16 Department of Molecular Medicine, Cleveland Clinic Lerner College of Medicine of Case Western Reserve University, Cleveland, OH USA

**Keywords:** Mitochondrial DNA, HIV, Cerebrospinal fluid, Neurocognitive impairment, Iron, Inflammation

## Abstract

**Background:**

Mitochondria are abundant organelles critical for energy metabolism and brain function. Mitochondrial DNA (mtDNA), released during cellular injury and as part of the innate immune response to viral pathogens, contains CpG motifs that act as TLR-9 ligands. We investigated relationships between cerebrospinal fluid (CSF) cell-free mtDNA levels and HIV viral load (VL), biomarkers of inflammation and iron transport, and neurocognitive (NC) function in the CNS HIV Antiretroviral Therapy Effects Research (CHARTER) cohort.

**Methods:**

We quantified cell-free mtDNA in CSF by droplet digital PCR in 332 CHARTER participants who underwent comprehensive neuropsychiatric evaluation. NC performance was assessed using the global deficit score (GDS) as either a continuous or a binary measure (GDS ≥ 0.5, impaired vs. GDS < 0.5, unimpaired). CSF, clinical, and biomarker data from the earliest available time point were analyzed. Cell-free mtDNA associations with CSF inflammation and iron-related biomarkers [CXCL10, IL-6, IL-8, TNF-a, transferrin (TF), ceruloplasmin (CP), and vascular endothelial growth factor (VEGF)], VL, and GDS were evaluated by multivariable regression.

**Results:**

CSF cell-free mtDNA levels were significantly lower in participants with undetectable (vs. detectable) VL in either plasma (*p* < 0.001) or CSF (*p* < 0.001) and in those on antiretroviral therapy (ART; *p* < 0.001). Participants on ART with undetectable VL in both CSF and plasma had lower mtDNA levels than those with detectable VL in both compartments (*p* = 0.001). Higher mtDNA levels were observed in participants in the highest vs. lowest tertile (T3 vs. T1) of CSF CXCL10 (T3 vs. T1, *p* < 0.001) and TNF-a (T3 vs. T1, *p* < 0.05) in unadjusted analyses. MtDNA levels also correlated with CSF leukocyte count. After adjusting for CSF leukocyte count and VL, mtDNA levels were also associated with other inflammation- and iron-related biomarkers in CSF, including TF (T3 vs. T1, *p* < 0.05) and CP (T3 vs. T1, *p* < 0.05). With additional correction for ART use, mtDNA was also negatively associated with CSF VEGF (*p* < 0.05) and IL-6 (*p* = 0.05). We observed no associations of CSF mtDNA levels with age or GDS-defined NC impairment.

**Conclusions:**

CSF cell-free mtDNA levels were associated with HIV RNA and ART status, as well as with biomarkers of iron transport and VEGF, a growth factor with known effects on mitochondrial integrity and autophagy. CSF mtDNA may be a biomarker of iron dysregulation and/or neuroinflammation during HIV infection.

**Electronic supplementary material:**

The online version of this article (doi:10.1186/s12974-017-0848-z) contains supplementary material, which is available to authorized users.

## Background

The pathogenesis of HIV-associated neurocognitive impairment (NCI) remains incompletely understood. Neuroinflammation due to chronic immune activation and viral replication in the central nervous system (CNS) is important in promoting HIV-associated NCI (reviewed in [1] and [2]). In individuals with effective viral suppression on antiretroviral therapy (ART), other risk factors may contribute, including metabolic disorders, cardiovascular risk factors, and older age [3]. Notably, mitochondrial dysfunction is associated with all of these factors [4].

Mitochondria are intracellular organelles, critical for energy production and a host of metabolic processes, which contain their own distinct DNA genome (mtDNA). Most mammalian cells contain multiple copies of mtDNA (between 100 and 10,000 copies, depending upon cell type and function) [5, 6]. Brain tissue, by virtue of its high bioenergetic needs, contains higher mtDNA copy numbers than most other tissues [6]. Given the endosymbiotic origins of mitochondria, mtDNA has similarities with bacterial genomes, including high numbers of CpG motifs. These motifs tag free mtDNA as a “damage-associated molecular pattern” molecule (DAMP), which triggers toll-like receptor (TLR)-9 activation and downstream inflammation [7].

Mitochondrial damage and mtDNA copy number within brain tissue have been associated with several neurodegenerative diseases, as well as with aging [8–10]. Fewer copies of mtDNA and larger numbers of mtDNA mutations are found in the brains of individuals with Parkinson’s disease (PD) and Alzheimer’s disease (AD), suggesting a possible role for reduced mitochondrial function in causing or promoting PD and AD neuropathology [11]. More recently, investigators have begun exploring the clinical utility of measuring cell-free mtDNA in cerebrospinal fluid (CSF) as a biomarker of mitochondrial damage or dysfunction and associated inflammation in the brain. In children with traumatic brain injury, higher cell-free CSF mtDNA levels are associated with severe disability and death [12]. Furthermore, cell-free mtDNA levels are lower in individuals with AD and PD when compared to healthy controls or individuals with other neurodegenerative diseases [13, 14].

Disruption of iron homeostasis is also a characteristic feature of neurodegenerative disorders, including AD and PD [15–17], and iron transport and iron homeostasis are required for mitochondrial biogenesis and function [18]. Furthermore, either iron accumulation or iron depletion can promote free radical formation, which may then also induce inflammation, mitophagy, and neurodegeneration [19–22]. In this study, we examined the relationship between CSF cell-free mtDNA and CSF biomarkers of inflammation and iron transport, HIV disease-related factors, and NCI in participants enrolled in the CNS HIV Antiretroviral Therapy Effects Research (CHARTER) study. We hypothesized that CSF levels of iron biomarkers and cell-free mtDNA, both of which are related to mitochondrial integrity, could explain the presence and the degree of NCI in HIV-infected individuals.

## Methods

### Study population and design

CHARTER is a prospective, observational study that enrolled ambulatory, HIV-infected adults from 2003 to 2007 at six USA locations: Johns Hopkins University, Baltimore, MD; the Icahn School of Medicine at Mount Sinai, New York, NY; University of California, San Diego, CA; University of Texas Medical Branch, Galveston, TX; University of Washington, Seattle, WA; and Washington University, St. Louis, MO. Standardized, comprehensive neuromedical, neurobehavioral, and laboratory assessments were performed, as previously described [23, 24]. This study involved a cross-sectional analysis associating CSF biomarker measurements with clinical data in 332 CHARTER participants who had sufficient CSF from the baseline visit available for all measurements. The two most common antiretroviral regimens were a non-nucleoside reverse transcriptase inhibitor-based regimen (*n* = 107), and a protease inhibitor-based regimen (*n* = 152). The study was approved by institutional review boards at each site, and all participants provided written informed consent.

### Neurocognitive assessments

HIV-associated neurocognitive disorder (HAND) was diagnosed when NCI and functional impairment were determined to likely be due to HIV-related effects on the brain rather than to comorbid conditions [24]. Detailed review by a senior CHARTER neuropsychologist and neurologist, using published guidelines and the Frascati criteria [25], provided categorization of comorbid conditions for all CHARTER participants as incidental to, contributing to, or confounding their neurocognitive performance. Several conditions (e.g., brain trauma, epilepsy or other seizure history, CNS opportunistic diseases) informed this categorization; detailed information on their frequencies are presented elsewhere [23]. Individuals with confounding neurocognitive comorbidities (15% of the total CHARTER cohort), which by definition precluded an assessment of the contribution of HIV infection to their neurocognitive impairment, were not eligible for a diagnosis of HAND according to Frascati criteria [23] and were excluded from the present study. The global deficit score (GDS), calculated by averaging deficit scores from a battery of neurocognitive tests, was also used as a measure of neurocognitive performance, with NCI defined as GDS ≥ 0.5 [26]. Follow up neurocognitive assessments were available on 216 of our 332 subjects. We categorized these 216 individuals as improved, stable, or declined based on comparison between the baseline and last follow-up assessment (See Additional file 1: Table S1).

### Clinical laboratory assessment

HIV infection was diagnosed by ELISA with western blot confirmation. HIV viral load (viral RNA copies per ml) were measured in plasma and CSF by reverse transcriptase PCR (Roche Amplicor, v. 1.5, lower limit of quantitation 50 copies/mL) performed at a central lab [23].

### Cell-free mtDNA analysis

CSF samples, obtained at the baseline visit, were analyzed for cell-free mtDNA content. Briefly, CSF was centrifuged at 250×*g* for 15 min to remove cells and cellular debris, and the supernatant was stored at −80 °C. Total genomic DNA was extracted from CSF supernatant samples using QIAamp DNA Mini Kit (Qiagen, CA) according to the manufacturer’s protocol, and was digested using the BamHI HF (New England Biolabs, Ipswich, MA) restriction enzyme. MtDNA was then quantified by droplet digital PCR (Bio-Rad, Hercules, CA) using a primer-probe combination targeting the NADH dehydrogenase 2 gene (MT-ND2, Applied Biosystems, Foster City, CA) found within the mitochondrial genome. A genomic DNA (gDNA) control was also measured by targeting the ribonuclease P protein subunit p30 gene (RPP30, Applied Biosystems, CA). Quantification was performed according to manufacturer’s instructions and was carried out in triplicate in 20 μL reactions, consisting of 10 μL of 2× Bio-Rad supermix for probes, 1 μL of either 20× Primer/FAM ND2 mix or 20× Primer/VIC RPP30 mix, 4 μL of molecular grade water, and 5 μL of DNA. Droplet generation and amplification were performed per protocol, and after analysis of the droplets, mtDNA copy number per mL of CSF was calculated using QuantaSoft (Bio-Rad, CA).

### Measurement of other biomarkers

Concentrations of inflammation and angiogenesis biomarkers were measured in CSF according to manufacturer’s protocol by commercial high-sensitivity multiplex (interleukin (IL)-6, IL-8, tumor necrosis factor-alpha (TNF-α) or single-plex (CXC motif chemokine 10 (CXCL10), vascular endothelial growth factor (VEGF) bead-based immunoassay arrays (Luminex FlexMap 3D platform, Millipore, Billerica, MA). Ten percent of all assays were repeated to assess operator and batch consistency.

Quantification of CSF iron content was performed using the Quantichrom Iron Assay (BioAssay Systems, Hayward, CA), following the manufacturer’s protocol. CSF transferrin content was determined by enzyme-linked immunoabsorbent assay (ELISA, Human Transferrin ELISA kit (ab108902), Abcam, Cambridge, MA), performed according to the manufacturer’s protocol. CSF samples were centrifuged, and duplicate samples were diluted 1:1000 with mix diluent. The human heavy chain ferritin (H-ferritin) content of CSF samples was determined by ELISA assay (Human H-Ferritin ELISA kit, Abnova, Taipei, Taiwan) performed according to the manufacturer’s protocol. Additional iron-transport-related biomarkers quantified in CSF included ceruloplasmin (CP) and haptoglobin (HP). Levels of these proteins were measured using commercially available bead-based immunoassays (Millipore) validated for CSF.

### Statistical analyses

Univariate linear regressions were performed to assess associations between mtDNA levels and clinical variables, including demographic characteristics, ART use, CSF HIV viral load and biomarkers of inflammation, angiogenesis, and iron transport in CSF. To fulfill the normality assumption of linear regression, mtDNA copy number was log-transformed. CSF biomarkers other than mtDNA, which were also continuous but non-normally distributed variables, were categorized as tertiles to improve sensitivity of statistical association tests. Student’s *t* test was used to determine the difference in log mtDNA levels between participants who were on ART and those who were not on ART. Within the ART-treated subset, analysis of variance (ANOVA) was used to determine differences in mtDNA levels between the following four groups: (1) detectable plasma and CSF viral load (*n* = 27), (2) detectable CSF viral load but undetectable plasma viral load (*n* = 5), (3) detectable plasma viral load but undetectable CSF viral load (*n* = 56), and (4) undetectable plasma and CSF viral load (*n* = 147). The small number of subjects with detectable HIV in CSF but not plasma limited the power to compare this category with other groups. Pearson’s correlations were calculated to evaluate the relationship between mtDNA level and CSF leukocyte count. To evaluate the relationship between mtDNA and neurocognitive performance, Wilcoxon tests were performed to compare mtDNA levels between the individuals with no NCI, and those with asymptomatic NCI (ANI) and mild NCI or neurocognitive disorder (MND). Too vfew participants had HIV-associated dementia (HAD, *n* = 5) to power comparisons with the other groups. In order to determine if associations with mtDNA were independent of other factors that may relate to neuroinflammation and NCI, associations between cell-free mtDNA levels and neurocognitive performance were assessed in multivariable linear regression models, stratified by ART status (on vs*.* off) and adjusted for nadir CD4+ T-cell count, CSF leukocyte count, and CSF virus detectability, or adjusted for nadir CD4+ T-cell count, CSF leukocyte count, and plasma virus detectability [27, 28]. Similar models were also used to assess associations of cell-free mtDNA levels with other CSF biomarkers (iron, ferritin, transferrin, CP, HP, IL-6, IL-8, CXCL-10, TNF-α, and VEGF) as independent outcomes, stratifying by ART status and adjusting for CSF WBC, in case differences in CSF mtDNA were driven by pleocytosis (WBC elevations in the CSF that occur as a result of CNS infections). These linear regression models were also run with CSF virus detectability included as a covariate. Statistical analyses were performed using R version 3.1.2 [29] and SAS 9.4. Two-sided *p* values <0.05 were considered statistically significant.

## Results

### Study population

The mean age of the study population was 43 years (standard deviation 8.2); 77% were men, and 54% reported non-white race/ethnicity. Sixty-nine percent of participants were on ART at the time of CSF sampling. The median HIV RNA concentrations were 1.9 in plasma (Interquartile range (IQR) 1.7–4.0) log_10_ copies/ml and 1.7 in CSF (IQR 1.7–2.3) log_10_ copies/ml. Thirty-four percent (plasma) and 60% (CSF) had undetectable HIV RNA. The median (IQR) current CD4^+^ T-cell count was 455 (270–636) cells/mm^3^. Thirty percent of participants were classified as having NCI based on the GDS [26], and these impaired participants were slightly older than those without NCI (45 vs. 43 years, *p* = 0.02).Unimpaired participants were also less likely to have a self-reported diagnosis of diabetes (Type I or II) (5 vs. 20%, *p* = 0.001). Demographic and clinical characteristics of study participants are presented, overall and stratified by NCI status, in Table 1.

**Table 1:** Study participant characteristics broken down by the presence or absence of HIV-associated neurocognitive impairment as defined by global deficit score

Variable (n, %, or median, IQR)	Overall (*n* = 332)	NP-unimpaired (*n* = 234)	NP-impaired (*n* = 98)	*p* value
Age (years)	43.0 (38.8–48.0)	43.0 (38.0–48.0)	45.0 (40.3–49.8)	0.019
Education (years)	12 (11–14)	12 (11–14)	13 (12–16)	0.030
Self-reported race/ethnicity (*n*, %)				
Black	138 (42%)	107 (46%)	31 (32%)	
Hispanic	30 (8%)	21 (9%)	12 (12%)	
White	152 (46%)	99 (42%)	53 (54%)	
Other	9 (3%)	7 (3%)	2 (2%)	0.095
Sex (*n*, % male)	262 (77%)	184 (79%)	78 (80%)	0.883
Diabetes mellitus (*n*, %)	31 (10%)	12 (5%)	19 (20%)	0.001
BMI	25.8 (23.2–29.0)	25.5 (23.1–29.3)	26.3 (24.1–28.6)	0.437
Plasma HIV RNA (log_10_ copies/ml)	1.9 (1.7–4.0)	2.2 (1.7–4.1)	1.7 (1.7–3.5)	0.087
Undetectable HIV RNA in plasma (*n*, %)	113 (34%)	79 (34%)	34 (34%)	0.899
CSF HIV RNA (log_10_ copies/ml)	1.7 (1.7–2.3)	1.7 (1.7–2.4)	1.7 (1.7–1.9)	0.164
Undetectable HIV RNA in CSF (*n*, %)	198 (60%)	132 (56%)	64 (65%)	0.144
Absolute CD4+ T-cell count	455 (270–636)	441 (264–645)	461(308–632)	0.639
CD4^+^ T-cell nadir	193 (60–315)	204 (69–325)	174(57–281)	0.224
HCV status negative (*n*, %)	78 (24%)	54 (23%)	24 (24%)	0.778
ART status (*n*, %)				
ART-naïve	51 (15%)	38 (16%)	13 (13%)	
ART	230 (69%)	158 (67%)	72 (74%)	
Non-ART	44 (13%)	34 (15%)	10 (10%)	
No ARVs	7 (2%)	4 (2%)	3 (3%)	0.508
WRAT	96 (85–105)	97(85–107)	94(83–102)	0.143
Beck’s Depression Inventory Score	11(5–22)	11(5–20)	12(6–24)	0.399

Diabetes mellitus was self-reported and could be either type I or II

*IQR* interquartile range, *ART* highly active antiretroviral therapy, *BMI* body mass index, *HCV* hepatitis C virus, A*RVs* antiretroviral drugs, *WRAT* Wide Range Achievement Test, *CSF* cerebrospinal fluid, *BMI* body-mass index, *NP* neurocognitive performance

### Socio-demographic and clinical factors associated with cell-free CSF mtDNA levels

**Fig. 1:**
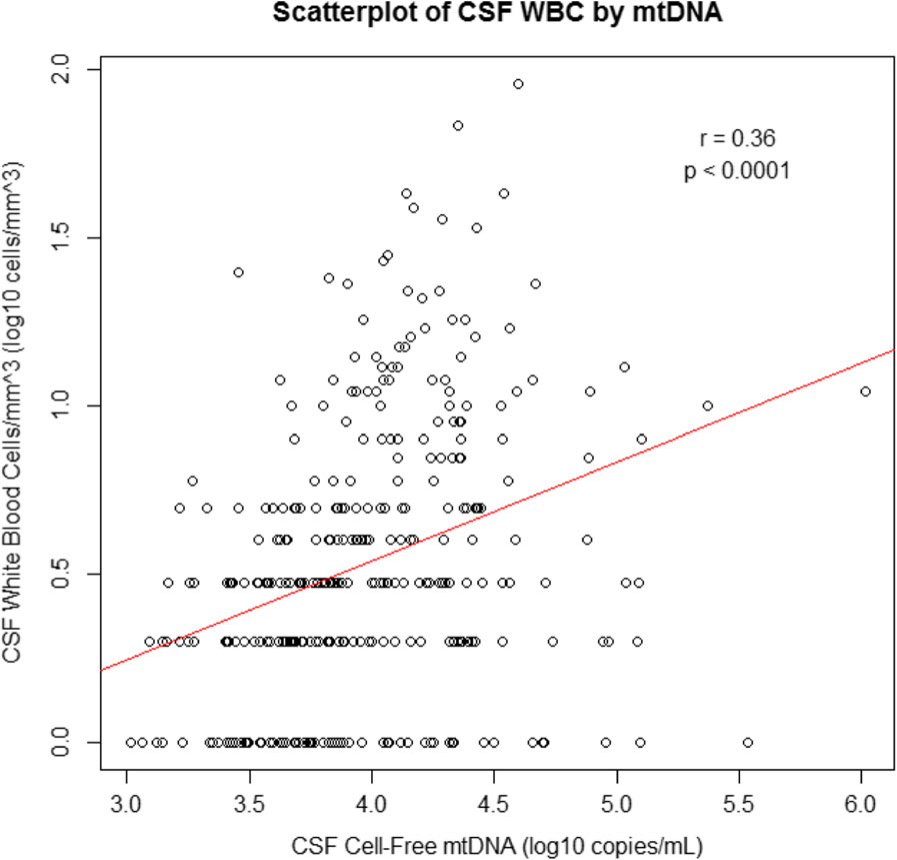
Scatterplot of CSF WBC by mtDNA. CSF cell-free mtDNA (log10 copies/ml) plotted against CSF white blood cells/mm^3^

Overall median (IQR) CSF cell-free mtDNA was 3.92 (3.67–4.27) log_10_ copies/ml. Individuals on ART at the time of CSF sampling had significantly lower cell-free CSF mtDNA levels than those who were ART-naïve (*p* = 0.03) or who had previously received ART (*p* = 0.02). Additionally, participants with detectable HIV RNA in either plasma or CSF (grouped together) had significantly higher cell-free CSF mtDNA levels than those with undetectable HIV RNA in either plasma (*p* < 0.001) or CSF (*p* < 0.001) *(data not shown)*. Higher cell-free CSF mtDNA was seen with lower nadir CD4+ T-cell counts (*p* < 0.01). Higher CSF WBC and CSF HIV RNA were also associated with higher mtDNA levels (both *p* < 0.001), with a modest positive correlation between CSF WBC and cell-free CSF mtDNA (*r* = 0.36, *p* < 0.001; Fig. 1). This association also persisted in individuals both on and off ART. After adjusting for CSF HIV RNA and nadir CD4+ T-cell count, women had significantly lower median cell-free CSF mtDNA levels than men (*p* = 0.01). No associations between CSF cell-free mtDNA levels and age, self-reported race/ethnicity, body-mass index, lifetime diagnosis (self-report) of diabetes mellitus, or depression (Beck’s Depression Inventory Score) were identified.

### Association of cell-free CSF mtDNA levels with neurocognitive performance

**Fig. 2:**
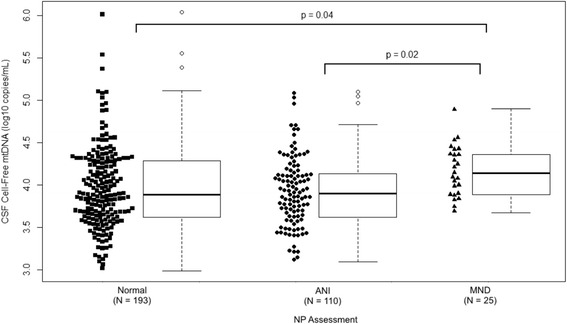
CSF mtDNA in relation to neurocognitive status. CSF cell-free mtDNA levels in relation to neurocognitive status as measured by Frascati criteria. Since only five participants had HIV-associated dementia, they were not included in this analysis. (*ANI* asymptomatic neurocognitive impairment, *MND* mild neurocognitive impairment or disorder)

No association was observed between CSF mtDNA levels and GDS-defined NCI. However, CSF mtDNA was significantly higher in 25 participants with MND compared to those without NCI (*n* = 193, 4.17 vs. 3.97 log_10_ copies/ml; *p* = 0.04) or to those with ANI (*n* = 110, 4.17 vs. 3.93 log_10_ copies/ml; *p* = 0.02). No individuals with MND had a mtDNA level less than 3.69 log_10_ copies/ml (Fig. 2).

We did not observe any association between the CSF mtDNA level and the trajectory of neurocognitive status, as *t* tests comparing the improved/stable vs. declined, and improved vs. declined groups showed no significant differences (*p*V values of 0.96 and 0.73, respectively).

### Inflammation, angiogenesis, and iron biomarker associations with cell-free CSF mtDNA

In univariate analyses (*n* = 332) comparing CSF mtDNA levels in the third (T3) and first (T1) tertiles of each biomarker, mtDNA levels were positively associated with CXCL-10 (T3 vs. T1, *p* < 0.001) and TNF-α (T3 vs. T1, *p* = 0.04) (Additional file 1: Table S2). No significant associations of mtDNA levels were seen with CSF iron, ferritin, transferrin, CP, HP, or other biomarkers, including VEGF, in unadjusted analyses. To better understand the independent contribution of cell-free mtDNA to inflammation and iron transport in the CNS, analyses of mtDNA were adjusted for the potentially confounding effects of HIV RNA, and CSF WBC. After adjustment for these covariates (*n* = 329), lower cell-free CSF mtDNA levels were associated with higher levels of CSF transferrin (T3 vs. T1; OR 0.69, *p* = 0.01), and CP (T3 vs. T1; OR 0.71, *p* = 0.02). Age did not contribute to any of these models. Associations with CXCL-10 and TNF-α did not persist after adjustment for CSF WBC and HIV RNA.

We also addressed potential confounding due to non-suppressed HIV infection by analysis restricted to individuals on ART. In this subgroup (*n* = 294), CSF mtDNA was again negatively associated with CSF transferrin (*p* < 0.01), CP (*p* = 0.06), VEGF (*p* = 0.03), and IL-6 (*p* = 0.05). Since comorbid conditions can influence neurocognitive performance, we also stratified by the severity of comorbidities present. Among participants with comorbidities that were assessed to adversely affect neurocognitive performance, cell-free CSF mtDNA levels continued to be negatively associated with CSF transferrin (*p* = 0.02), CP (*p* < 0.01), VEGF (*p* < 0.05), and IL-6 (*p* < 0.05) (*n* = 134 except for iron biomarker levels (*n* = 80)), even after adjusting for CSF WBC. Among individuals who only had comorbidities deemed to be incidental, no associations with CSF mtDNA were identified.

## Discussion

Cell-free mtDNA is a novel biomarker being explored in inflammation [30, 31], cancer [32, 33], heart disease [34, 35], diabetes [31], and neurodegenerative processes [13, 14, 36]. These published data support a complex relationship in which higher cell-free mtDNA levels were associated with neuroinflammation, while lower levels were associated with neurocognitive impairment [13, 14]. In this CHARTER analysis, we determined associations of cell-free CSF mtDNA with CSF biomarkers of inflammation, angiogenesis, and iron transport, and with HIV-associated neurocognitive impairment, a phenotype likely driven by both inflammatory and neurodegenerative processes and in which iron transport may also be abnormal [37, 38]. We observed cell-free mtDNA levels in the range reported by other groups [13], and these levels were higher in individuals with detectable CSF HIV RNA and in the absence of ART. CSF mtDNA also increased with the levels of some (e.g., CXCL-10, TNF-α) but not all (e.g., IL-6) biomarkers of inflammation in univariate analyses. However, only the association with IL-6 persisted after adjusting for HIV RNA and CSF WBC, suggesting that these covariates may at least partially mediate the relationship between CSF mtDNA and inflammation. Finally, although mtDNA levels did not distinguish impaired from unimpaired individuals, they were higher in those with mild NCI as compared to asymptomatically impaired and unimpaired individuals, suggesting a link between CSF mtDNA, neuroinflammation, and NCI in HIV infection.

Interestingly, after adjusting for the presence of HIV RNA and WBCs in CSF, which are possible drivers of the release of, or the source of, cell-free mtDNA, we found that higher mtDNA levels were associated with lower levels of two proteins involved in iron metabolism and transport, CP and transferrin, and the angiogenesis marker (VEGF), supporting novel mechanisms of CNS injury in HAND. CP plays a critical role through its ferroxidase activity which facilitates binding of iron to transferrin. In addition, both CP and VEGF have neuroprotective as well as strong angiogenic properties [39, 40]. CP and VEGF are also acute-phase proteins and may themselves reflect levels of neuroinflammation. However, the absence of positive associations of cell-free mtDNA levels with other measured inflammation markers in multivariable-adjusted analyses suggests that, in the absence of HIV replication in the CSF and CSF pleocytosis, increased mtDNA levels primarily reflect disrupted CNS iron transport in HIV-infected adults. For example, CP is expressed primarily in a membrane-bound form by astrocytes, pericytes, and brain endothelial cells to provide the ferroxidase activity needed for oxidation of ferrous iron that is released into the brain and bound to transferrin [41, 42]. Downregulation of CP can therefore lead to cellular iron accumulation, mitochondrial injury, and oxidative stress [42]. Likewise, lower levels of VEGF may have adverse effects, since this molecule has beneficial effects on mitochondrial integrity, and it inhibits autophagy [43]. Consistent with this interpretation, transferrin, which tends to decline in the serum during inflammation but is essential for iron transport across the blood-brain barrier [44], was also negatively associated in CSF with mtDNA (when controlling for CSF HIV RNA and WBC). Altered CSF transferrin levels may indicate disrupted iron transport in the CNS, perhaps leading to mitochondrial degeneration, as cellular iron is an important co-factor for mitochondrial biogenesis [18]. In contrast to other biomarkers of inflammation, lower CSF IL-6 levels were consistently associated with higher mtDNA in individuals on ART. Although IL-6 is a well-known pro-inflammatory cytokine, it is also important in regulation of iron metabolism and macrophage iron content via the hepcidin-ferroportin axis [38]; hence, this association may relate mainly to its iron-regulatory functions. Inverse associations of all of these biomarkers with cell-free mtDNA therefore point to a disruption of iron homeostasis and induction of neuroprotective mechanisms in HIV-infected individuals, which may in turn be linked to altered mtDNA biogenesis and/or mitophagy in HAND [19, 45].

In persons with HIV RNA in the CSF, pleocytosis was more common than in those with suppressed HIV replication on ART [46–48], and in the present study, cell-free mtDNA levels correlated with CSF WBC as well as with CSF HIV RNA. The source of this mtDNA remains unclear, as mtDNA may be released by dying cells, or in neutrophil extracellular traps [49]. There may also be reduced mtDNA degradation as neurons depend on astrocytes for mitochondrial degradation (a process termed transmitophagy) [50]. Individuals chronically infected with HIV have impaired astrocyte function, leading to impaired clearance of neurotransmitters and other molecules through disrupted CNS proteostasis (reviewed in [51, 52]). In addition, the reduced CP association with higher CSF mtDNA may reflect sequestration of iron by astrocytes [53]. On the other hand, antiretroviral drugs, particularly those drugs with good CNS penetration, generally reduce CNS inflammation, and therefore, the association between ART and reduced CSF cell-free mtDNA is expected. However, some ART drugs (e.g., certain nucleoside analogues) can reduce cellular levels of mtDNA due to interference with mtDNA synthesis, and this observation could provide an alternative explanation for reduced CSF mtDNA levels in persons on ART.

Limitations of this study include the cross-sectional design, some degree of heterogeneity of treatment regimens in cohort participants on ART, and inability to extrapolate from cell-free mtDNA levels in CSF to intracellular mtDNA content in the brain. Nevertheless, this study provides new insights into the complex pathophysiology of neuroinflammation and mitochondrial damage during HIV infection.

## Conclusions

In conclusion, cell-free CSF mtDNA was inversely associated with biomarkers of iron transport in HIV-infected persons when controlling for CSF HIV RNA and WBCs. Given the importance of iron homeostasis in neuronal function [54] and also in regulating mitochondrial biogenesis, the relationship between mtDNA and markers of iron homeostasis [18] may reflect HIV-associated dysregulation of iron transport in the brain, including the possibility of iron deficiency in neurons and iron overload in astrocytes. This study is the first to report these specific associations in HIV-infected persons. These findings may have implications for the etiology of persistent neuroinflammation and neurodegeneration in HIV-infected persons and merit further investigation.

## Additional file

Additional file 1: Table S1. Categorization of the 216 individuals with follow-up neurocognitive assessments. Baseline assessments were compared to the assessment at the last follow-up visit, and individuals were categorized as improved, stable, or declined.  **Table S2.** Univariate analyses of log CSF-free mtDNA and CSF biomarkers of inflammation and iron transport. (DOCX 12 kb)

## Abbreviations

AD: Alzheimer’s disease
ANI: Asymptomatic neurocognitive improvement
ANOVA: Analysis of variance
ART: Antiretroviral therapy
CHARTER: CNS HIV Antiretroviral Therapy Effects Research
CNS: Central nervous system
CP: Ceruloplasmin
CSF: Cerebrospinal fluid
CXCL10: CXC motif chemokine 10
DAMP: Damage-associated molecular pattern
gDNA: Genomic DNA
GDS: Global deficit score
HAD: HIV-associated dementia
HAND: HIV-associated neurocognitive disorder
H-ferritin: Human heavy chain ferritin
HP: Haptoglobin
IQR: Interquartile range
MND: Mild neurocognitive disorder
mtDNA: Mitochondrial DNA
NC: Neurocognitive
NCI: Neurocognitive impairment
PD: Parkinson’s disease
TF: Transferrin
TLR: Toll-like receptor
VEGF: Vascular endothelial growth factor
VL: HIV viral load
