# Supplementary material for: Cerebrospinal fluid cell-free mitochondrial DNA is associated with HIV replication, iron transport, and mild HIV-associated neurocognitive impairment
Source: J Neuroinflammation. 2017 Mar 31;14:72. doi: 10.1186/s12974-017-0848-z (PMC5374652; doi:10.1186/s12974-017-0848-z)
Supplement: Additional file 1: Table S1. — Categorization of the 216 individuals with follow-up neurocognitive assessments. Baseline assessments were compared to the assessment at the last follow-up visit, and individuals were categorized as improved, stable, or declined. Table S2. Univariate analyses of log CSF-free mtDNA and CSF biomarkers of inflammation and iron transport. (DOCX 12 kb) [file 12974_2017_848_MOESM1_ESM.docx]

| Final NC change status at last follow-up visit | |
| --- | --- |
| improved | 34 |
| stable | 137 |
| declined | 45 |
| total | 216 |

**Table S1**. Categorization of the 216 individuals with follow-up neurocognitive assessments. Baseline assessments were compared to the assessment at the last follow-up visit, and individuals were categorized as improved, stable, or declined.

| **Parameter** | **Estimate** | **t-Value** | **P-Value** |
| --- | --- | --- | --- |
| Ceruloplasmin (T3 vs T1) | -0.207 | -1.49 | 0.1365 |
| Haptoglobin (T3 vs T1) | -0.109 | -0.13 | 0.8929 |
| IL-6 (T3 vs T1) | 0.023 | 0.17 | 0.8662 |
| IL-8 (T3 vs T1) | 0.171 | 1.24 | 0.2145 |
| IP-10 (T3 vs T1) | 0.494 | 3.69 | 0.0003 |
| TNF-α (T3 vs T1) | 0.281 | 2.07 | 0.0391 |
| VEGF (T3 vs T1) | -0.130 | -0.88 | 0.3774 |
| Iron (T3 vs T1) | -0.117 | -0.84 | 0.3993 |
| Ferritin (T3 vs T1) | -0.031 | -0.25 | 0.8010 |
| Transferrin (T3 vs T1) | -0.212 | -1.51 | 0.1309 |

**Table S2.** Univariate Analyses of log CSF free mtDNA and CSF Biomarkers of Inflammation and Iron Transport
